# Splenic Infarction in a Patient With Sickle Cell Trait Following High-Altitude Exposure

**DOI:** 10.7759/cureus.77438

**Published:** 2025-01-14

**Authors:** Eian G Prohl, Neel K Vaidya

**Affiliations:** 1 Internal Medicine, Veterans Affairs Greater Los Angeles Healthcare System, Los Angeles, USA; 2 Internal Medicine, David Geffen School of Medicine at the University of California Los Angeles, Los Angeles, USA; 3 Radiology, Veterans Affairs Greater Los Angeles Healthcare System, Los Angeles, USA

**Keywords:** high altitude illness, ie splenic infarct, rare cause of acute abdominal pain, sickle cell disorders, sickle cell trait

## Abstract

Sickle cell trait (SCT) is a generally asymptomatic carrier condition but clinical complications are recognized. Splenic infarction from exposure to high altitude is one complication of sickle cell trait. We report a 53-year-old man who traveled to Mammoth Lakes, California, and experienced an onset of abdominal pain approximately seven hours after arrival. In the local emergency department, the patient was diagnosed with presumed SCT using the sickle cell solubility test. Contrast-enhanced computed tomography (CECT) of the abdomen and pelvis obtained 12 hours after symptom onset was read as mild heterogenous enhancement of the spleen but without acute disease. After supportive care, the patient was discharged the next day with recommendations to descend immediately for presumed splenic syndrome. When his symptoms continued for days unabated, the patient presented to his primary care office, and repeat imaging performed 10 days later demonstrated a large splenic infarction. The patient recovered fully with supportive therapy and the diagnosis of SCT was confirmed by hemoglobin electrophoresis.

## Introduction

In the United States, approximately 3 million persons live with SCT, including an estimated 7% of the African American population [1,2]. Only since 2006 have all U.S. states conducted universal newborn screening for sickle cell disease [3]. Sickle cell trait cannot be detected by measuring hemoglobin, hematocrit, reticulocyte count, or other red blood cell indices. Not surprisingly, many adults with SCT are unaware of their status [4,5]. Expert opinion suggests offering sickle cell trait screening to adults regardless of ethnicity when they may benefit from the information, including individuals planning pregnancy or intense physical exertion, especially at high altitudes [6]. In addition to providing preliminary information on the reproductive consequences of SCT and considering referral to a genetic counselor, patient education should convey that during travel to high altitude or times of intense exertion, vaso-occlusive phenomenon, and rhabdomyolysis have been described, and that increased rates of venous thromboembolism (VTE) and a rare form of renal carcinoma are associated with SCT [1,6]. This in turn provides an opportunity to reinforce universal healthy practices and precautions while emphasizing that SCT is not a disease. These precautions include adequate hydration, avoidance of sustained intensive exercise in high heat, proper acclimatization to high altitudes, awareness of VTE risk from prolonged immobility, and promptly seeking treatment for acute abdominal pain or hematuria. If individuals do not want to know their carrier status, they can still be given these universal precautions.

Splenic syndrome refers to acute left upper quadrant abdominal pain and tenderness with or without splenomegaly, postulated to occur due to sickled red cells causing vaso-occlusive infarction and/or splenic sequestration [7]. Reports of a splenic syndrome associated with exposure to high altitude in sickle cell trait were first published in the early 1950s following flights of Black American servicemen in unpressurized aircraft above 3,000 meters [8,9]. Numerous corroborating reports were published in the following decades [10-16].

## Case presentation

**Figure 1:**
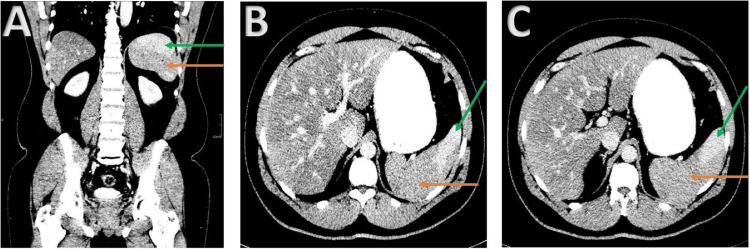
Contrast enhanced computed tomography (CECT) images obtained 12 hours after symptom onset Coronal (A) and axial (B and C) CECT images in the portal venous phase demonstrate abnormal enhancement of the spleen with a large non-enhancing area within the posterior and inferior spleen (orange arrows). Normal enhancing spleen is noted anteriorly (green arrows).

A 53-year-old African-American male with a past medical history notable for type 2 diabetes mellitus and severe obstructive sleep apnea intolerant of positive airway pressure therapy traveled for a vacation to Mammoth Lakes, California from his home near sea level in Los Angeles, California while in his usual state of health. Upon arrival at 19:00 to his accommodations at an elevation of 2,700 meters above sea level, he noted shortness of breath upon exertion but no other symptoms. At an evening dinner banquet, he danced before returning to his accommodations and falling asleep around midnight. Around 01:00, he was awoken from sleep with severe diffuse abdominal pain, bloating, and nausea. He also noted worsening shortness of breath and felt dyspneic at rest. He presented to a local emergency department (elevation 2450 m) at 06:00 where initial vital signs showed a temperature of 37.1֯ C, pulse of 105 beats per minute, respiratory rate of 16 breaths per minute, blood pressure 158/113 mm Hg, and oxygen saturation of 92% on room air. His physical examination was notable for diffuse abdominal tenderness with maximal tenderness in the left upper quadrant without organomegaly, rebound tenderness, or guarding. Home medications included escitalopram 10 mg daily, atorvastatin 20 mg daily, pioglitazone 30 mg daily, and semaglutide 1 mg weekly. Initial laboratory data is shown in Table 1. Contrast-enhanced computed tomography (CECT) of the abdomen and pelvis was obtained 12 hours after symptom onset (Figure 1). The radiologist noted mild heterogenous enhancement of the normal-sized spleen and concluded that no acute findings were present. A retrospective review of these images in liver window settings show a large non-enhancing area within the posterior and inferior spleen consistent with infarction. After treatment with supplemental oxygen, intravenous fluids, antiemetics, and opioid analgesia, the patient was discharged home 36 hours after symptom onset with the diagnosis of splenic syndrome due to high altitude exposure in the setting of SCT. He was advised that his pain should improve rapidly and resolve within a few days of descent.

**Table 1:** Laboratory results 7 hours, 3 days, and 10 days after symptom onset.

Test	Results 7 hours after symptom onset	Results 3 days after symptom onset	Results 10 days after symptom onset	Reference unit
White blood cells	13.5	10.2	6.26	4.5-11 k/uL
Red blood cells	4.15	4.6	4.48	4.6-6.2 M/uL
Hemoglobin	11.7	13.1	12	13.3-17.7 g/dL
Hematocrit	33.5	38.3	35.8	39-52 %
Mean corpuscular volume	80.7	82.8	79.9	80-99 fL
Red cell distribution width	13	13.9	12.5	12-15 %
Platelets	175	270	598	150-440 K/uL
Neutrophils	81	73.6	60	41-85 %
Lymphocytes	7	10.5	27	20-53 %
Eosinophils	0	0.3	0.8	0-6 %
Monocytes	11	15	9.4	5-15 %
Basophils	0	0	0.8	0-3 %
Immature granulocytes	0.4	0	-	0-1 %
Sodium	135	136	136	136-146 mmol/L
Potassium	4.3	4.4	4.6	3.5-5.3 mmol/L
Chloride	97	97	99	95-110 mmol/L
Carbon dioxide	30	29	26	24-31 mmol/L
Blood urea nitrogen	10	11	9	5-25 mg/dL
Creatinine	1.01	1	0.9	0.52-1.28 mg/dL
Glucose	293	245	281	70-110 mg/dL
Alanine transaminase	43	22	51	16-63 U/L
Aspartate aminotransferase	93	15	25	15-37 U/L
Alkaline phosphatase	84	88	73	33-94 U/L
Albumin	4	4.2	4	3.2-4.8 g/dL
Total bilirubin	1.3	0.7	0.5	0.2-1 mg/dL
Calcium	8.7	9.2	9.3	8.4-10.2 mg/dL
Lactate dehydrogenase	-	-	422	87-271 U/L
Ferritin	-	-	1277	22-322 ng/mL
Hemoglobin A1c	-	-	9.3	4.2-5.6 %
Sickle cell screen	POSITIVE	-	-	NEGATIVE

Upon arrival home, the patient’s abdominal pain and dyspnea persisted without improvement, so three days after symptom onset he returned to an emergency department near his home. A chest radiograph there showed a small left-sided pleural effusion and he was discharged with oral antibiotics for pneumonia treatment, without having repeat abdominal imaging performed. The documented physical exam was normal. Laboratory data for this visit are shown in Table 1.

**Figure 2:**
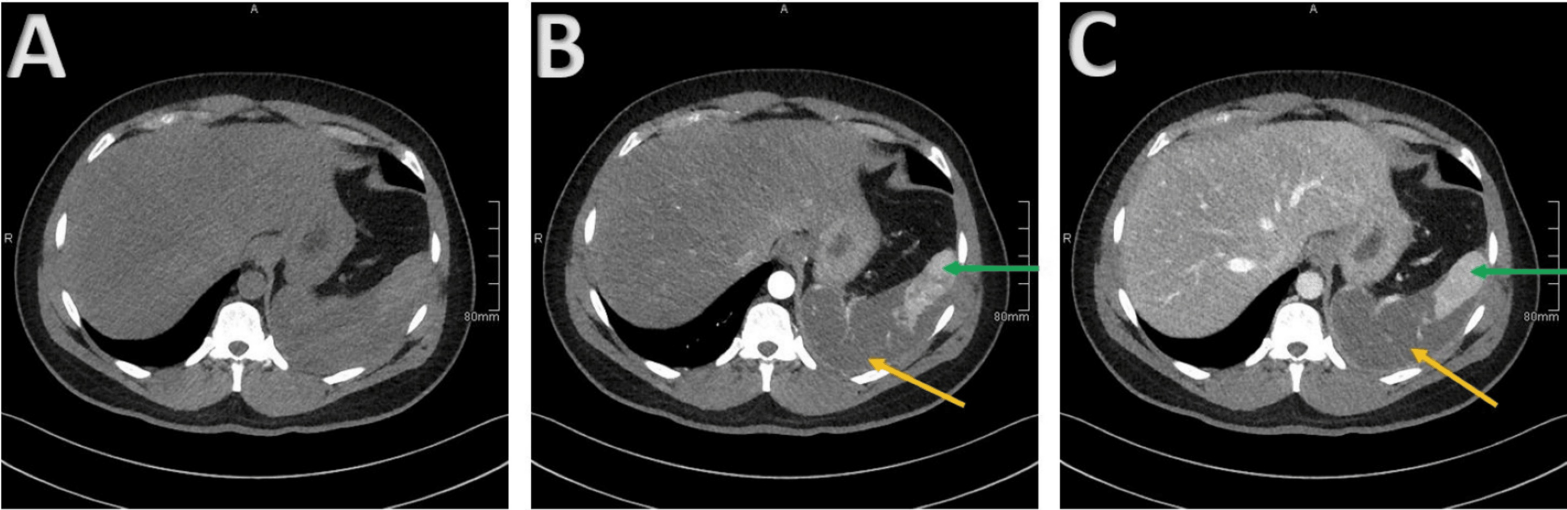
Computed tomography images obtained 10 days after symptom onset Axial non-enhanced (A) and contrast enhanced images in arterial phase (B) and portal venous phase (C) demonstrate a large non-enhancing area of the spleen (orange arrows) consistent with splenic infarction. Normal enhancing spleen is shown anteriorly (green arrows).

Ten days after symptom onset, the patient presented to his primary care physician for ongoing abdominal pain and bloating, while reporting near resolution of his dyspnea. On exam, vital signs were within normal limits. The abdominal exam showed distension with tenderness to palpation in the epigastrium and left upper quadrant. Laboratory data from this visit are also included in Table 1. He was directed to the emergency department for repeat abdominal imaging where a repeat CECT was consistent with a large splenic infarction (Figure 2).

After overnight observation and surgical consultation, the patient continued supportive outpatient treatment and recovered fully without splenectomy. His abdominal pain took approximately three months to fully resolve but was described as mild pain after four weeks. Hemoglobin electrophoresis was consistent with sickle cell trait, revealing 56.9% hemoglobin A, 0% hemoglobin-F, 2.6% hemoglobin A2, and 40.5% hemoglobin-S. At follow-up visits, he received recommended immunizations for functional asplenia in addition to education on his SCT status.

## Discussion

In the most comprehensive review of splenic infarction in SCT available, 54 case reports or case series of splenic infarction were found in the literature between 1970 and 2020, representing 85 cases, 75 (88%) of whom were male [17]. Whether the male preponderance of cases is due to a physiologic difference between sexes or a difference in rates of exposure to high altitude is unknown [14,17]. Of the 59 cases with altitude reported, two cases (3%) occurred under 1000 m, four cases (7%) occurred between 1001 and 2000 m, 17 cases (29%) occurred between 2001 and 3000 m, 27 cases (46%) occurred between 3001 and 4000 m, and two (3%) occurred above 4000 m, with the remainder reporting a range or ambiguous altitude levels [17]. A total of 29 individuals (34%) were physically active at onset of symptoms [17]. Goodman et al. found in their 1986-2006 case series that all 25 patients presented with abdominal pain and that vomiting, fever, and a palpable spleen were common. Elevated total bilirubin (95% of individuals), anemia (70%), and leukocytosis (50%) were the most common laboratory findings [14]. 

In this case, the highest previous altitude the patient reported visiting in his lifetime was approximately 1900 m and his activities there had included moderately strenuous exercise. His splenic infarction occurred at 2700 m, well within the range of altitudes frequently reported in other cases. His presenting abdominal pain, elevated total bilirubin, and anemia were typical. His dyspnea is a less common symptom and was likely due to irritation of the left hemidiaphragm. It is probable this patient’s comorbidities were contributory: his severe untreated sleep apnea would further exacerbate hypoxia while sleeping at elevation, and his hyperglycemia would have predisposed him to dehydration. 

Awareness of this SCT complication and a degree of suspicion in cases of abdominal pain following exposure to high altitude is important for timely diagnosis and correct management. The initial treating physician in the emergency department located at a high altitude likely had enhanced awareness of high-altitude medicine and correctly identified splenic syndrome in the setting of SCT as the likely diagnosis. Despite this, his large splenic infarction was not appreciated and the patient was given expectations for prompt resolution of symptoms with descent. 

Imaging obtained 12 hours after symptom onset was interpreted as nonspecific heterogeneity in enhancement, though in retrospect, portal venous phase images demonstrated a large non-enhancing area within the posterior and inferior spleen. Discussion with the reading radiologist to provide additional clinical context may be advisable when this diagnosis is suspected. Additionally, others have reported that initial CT findings in splenic infarction associated with SCT can be nonspecific and not predictive of the extent of splenic infarction, development of complications, or need for splenectomy [7,15]. Consideration of serial CT imaging has been recommended for these reasons [15].

Hayashi et al. reported that CT findings of non-segmental infarction (i.e., not corresponding to an arterial vascular territory) appear typical of high-altitude SCT cases and likely reflect the underlying microvascular occlusion [18]. Such findings are also occasionally referred to as splenic autoinfarction. This is in contrast to the wedge-shaped infarction seen in the most common cause of splenic infarction: a thromboembolic event [18,19]. Given this patient's SCT, antecedent high altitude exposure, and typical imaging findings of splenic infarction from microvascular occlusion, thromboembolic and thrombophilic workup was not felt to be warranted.

Most cases can be treated with supportive care [15,17,20]. Splenic abscess, rupture, hemorrhage, and chronic abdominal pain are potential complications for which surgical consultation is needed [15]. Upon discharge, close return precautions for fever, worsening pain, or dizziness that could herald these complications are advised. The typical duration of pain from splenic infarction in SCT is unclear, but one case series of six young military personnel suggested that 3-7 days was typical [15]. Our patient had more prolonged pain despite a lack of complications. Finally, recurrent splenic syndrome with repeat exposure to high altitude has been described [12,16]. Therefore, it may be prudent to advise avoidance of travel above their previous maximum tolerated altitude in cases not treated with splenectomy.

## Conclusions

Splenic infarction in SCT is a well-established but rare complication that occurs during travel above 2,000 meters in most cases. When patients present shortly after symptom onset, exam findings and imaging can be nonspecific, so the diagnosis requires a degree of suspicion. With time, symptoms and exams often point toward the spleen as the source of symptoms and CECT will show non-segmental or massive infarction. Treatment is supportive and splenectomy is usually not indicated. On discharge, patients should be given the expectation that resolution of symptoms may take weeks, but also receive strict return criteria for new or worsening symptoms. There should be a low threshold for repeat CECT in returning patients.

## References

[1] Naik RP, Smith-Whitley K, Hassell KL (2018). Clinical outcomes associated with sickle cell trait: A systematic review. Ann Intern Med.

[2] Ojodu J, Hulihan MM, Pope SN, Grant AM (2014). Incidence of sickle cell trait--United States, 2010. MMWR Morb Mortal Wkly Rep.

[3] Benson JM, Therrell BL Jr (2010). History and current status of newborn screening for hemoglobinopathies. Semin Perinatol.

[4] Treadwell MJ, McClough L, Vichinsky E (2006). Using qualitative and quantitative strategies to evaluate knowledge and perceptions about sickle cell disease and sickle cell trait. J Natl Med Assoc.

[5] Harrison SE, Walcott CM, Warner TD (2017). Knowledge and awareness of sickle cell trait among young African American adults. West J Nurs Res.

[6] Vichinsky EP (2024). Sickle cell trait. UpToDate.

[7] Murano T, Fox AD, Anjaria D (2013). Acute splenic syndrome in an African-American male with sickle cell trait on a commercial airplane flight. J Emerg Med.

[8] Cooley JC, Peterson WL, Engel CE, Jernigan JP (1954). Clinical triad of massive splenic infarction, sicklemia trait, and high altitude flying. J Am Med Assoc.

[9] Sullivan BH Jr (1950). Danger of airplane flight to persons with sicklemia. Ann Intern Med.

[10] Gonzalez L, Shapiro AF, Tafur A, Plaza-Meneses C, Sabando B (2020). Splenic infarct secondary to high altitude exposure in sickle cell trait patients: a case series. Cureus.

[11] O'Brien RT, Pearson HA, Godley JA, Spencer RP (1972). Splenic infarct and sickle-(cell) trait. N Engl J Med.

[12] Yanamandra U, Das R, Malhotra P, Varma S (2018). A case of autosplenectomy in sickle cell trait following an exposure to high altitude. Wilderness Environ Med.

[13] Scordino D, Kirsch T (2013). Splenic infarction at high altitude secondary to sickle cell trait. Am J Emerg Med.

[14] Goodman J, Hassell K, Irwin D, Witkowski EH, Nuss R (2014). The splenic syndrome in individuals with sickle cell trait. High Alt Med Biol.

[15] Pothula V, Saegusa E, Takekoshi D, Edson T, Ignacio R (2008). Splenic syndrome: a rare indication for splenectomy. Mil Med.

[16] Lane PA, Githens JH (1985). Splenic syndrome at mountain altitudes in sickle cell trait. Its occurrence in nonblack persons. JAMA.

[17] Jefferson JM, Sims WM, Umeh N (2021). Splenic infarction in sickle cell trait: A comprehensive systematic review of case studies. EJHaem.

[18] Hayashi TY, Matsuda I, Hagiwara K, Takayanagi T, Hagiwara A (2016). Massive splenic infarction and splenic venous thrombosis observed in a patient with acute splenic syndrome of sickle cell traits on contrast-enhanced thin-slice computed tomography. Abdom Radiol (NY).

[19] Antopolsky M, Hiller N, Salameh S, Goldshtein B, Stalnikowicz R (2009). Splenic infarction: 10 years of experience. Am J Emerg Med.

[20] Sheikha A (2005). Splenic syndrome in patients at high altitude with unrecognized sickle cell trait: splenectomy is often unnecessary. Can J Surg.

